# The Functional Role of Individual-Alpha Based Frontal Asymmetry in the Evaluation of Emotional Pictures: Evidence from Event-Related Potentials

**DOI:** 10.3389/fpsyt.2017.00180

**Published:** 2017-09-27

**Authors:** Tao Suo, Lei Liu, Chaoyang Chen, Entao Zhang

**Affiliations:** ^1^Institute of Psychology and Behavior, Department of Psychology, School of Education, Henan University, Kaifeng, China; ^2^Department of Psychology, Ningbo University, Ningbo, China

**Keywords:** frontal electroencephalography alpha asymmetry, emotional processing, resting electroencephalography, event-related potential, P300

## Abstract

The perceptual processing of emotional stimuli is subject to the regulation of brain function. This study investigated whether frontal electroencephalography (EEG) alpha asymmetry at resting conditions predicted the evaluation of emotional picture stimuli by event-related potentials (ERPs). In this study, participants first completed a 2-min resting task, and then passively viewed emotional pictures. The results showed that left active individuals had smaller frontal EEG alpha asymmetry scores to negative pictures than to positive and neutral pictures, whereas right active individuals had similar frontal EEG alpha asymmetry scores to negative, positive, and neutral pictures. Furthermore, the study showed a larger P300 to negative pictures than to positive and neutral pictures for left active individuals; however, there were no significant ERP differences to negative, positive, and neutral pictures for right active individuals. These findings suggest that frontal EEG alpha asymmetry at resting conditions can reflect interindividual differences in emotional perception tendencies to emotional picture stimuli.

## Introduction

The question what factors account for individual differences in the evaluation of emotional stimuli continues to be a central issue in the field of affective neuroscience. Research suggested that emotional processing was regulated by a functional lateralization of frontal cortex (1–5). A reliable correlate of frontal activity is frontal electroencephalography (EEG) alpha asymmetry that is reflected in the alpha frequency band (typically 8–13 Hz) measured with electroencephalography (EEG). Recent evidences suggested that there was an inverse relation between activity within the alpha range and cortical processing. For example, when underlying cortical systems was in active processing, the alpha tended to decrease (6–8). Thus, this study examined how frontal EEG alpha asymmetry was related to individual differences in the evaluation of emotional stimuli.

Frontal EEG alpha asymmetry includes two types of asymmetry: frontal EEG alpha asymmetry at resting conditions and frontal EEG alpha asymmetry during emotional challenge. The former is related this asymmetry to various trait-like individual differences and been referred to as trait frontal EEG alpha asymmetry; the latter is investigated in relation to manipulations that intended to influence emotional states and been labeled state frontal EEG alpha asymmetry (1, 2, 9). In line with a stable trait view, several studies suggested that frontal EEG alpha asymmetry in resting conditions can moderate the transient EEG asymmetry response to emotional stimuli. For instance, more left frontal EEG alpha asymmetry at resting conditions had been linked to superior emotional flexibility (4, 10), more effective emotion regulation (11, 12), lower stress-induced cortisol levels (5) as well as to less negative and more positive affect (13, 14). These studies suggest that individual frontal EEG alpha asymmetry at resting conditions can be used to predict the response to emotional stimuli.

Event-related potentials (ERPs) have high temporal resolution and can be used to study the unfolding of emotional processing. Especially, the P300 and late positive potential (LPP) are considered to be good neural indexes to track these stages of emotional processes (15–18). Researchers showed that P300 is sensitive to large number of cognitive processing, such as probability, task difficulty, and resource allocation (19, 20). In studies using emotional stimuli, the P300 has been summarized as reflecting “the allocation of capacity-limited resources toward motivationally salient environment stimuli,” in which motivationally relevant stimuli (e.g., emotional stimuli) naturally and automatically arouse and direct attentional resources (21, 22). Some research showed that emotional stimuli can attract our attention and produce larger P300 amplitude for emotional pictures compared to neutral pictures (17, 21, 23). Following P300 amplitude, a sustained positivity was observed, which was sensitive to the emotional stimuli. The LPP has been considered as a component related to the subjective evaluation of emotional stimuli. There was larger LPP amplitude for positive and negative pictures than neutral pictures (21, 24). However, no studies so far investigated whether frontal asymmetry predicted the evaluation of emotional picture stimuli using ERPs.

This study aimed to examine whether frontal EEG alpha asymmetry at resting conditions predicted the evaluation of emotional picture stimuli by ERPs. For the purposes of this study, we were interested in the P300 and LPP response as they related to processing of emotion stimuli. In this study, participants first completed a 2-min resting task, and then passively viewed emotional pictures. We expected that the transient EEG asymmetry response to emotional picture stimuli would be moderated by frontal EEG alpha asymmetry at resting conditions; we also expected left frontal EEG alpha asymmetry group, not right frontal EEG alpha asymmetry group, would appear different neural responses to emotional picture stimuli in the P300 as well as LPP.

## Materials and Methods

### Participants

Thirty-six undergraduates (25 females, 19–32 years old, M = 22.44 years, SD = 2.71 years) were recruited to participate in this study. All participants were right-handed with normal or corrected-to-normal vision and had no psychological or neurological disorders. All participants gave their written informed consent, and the study was approved by the local Ethics Committee of Henan University.

### Stimuli Material

Emotional pictures were chosen from IAPs based on the ratings of arousal and valence. There were 62 pictures in each of the following three groups: positive pictures, negative pictures, and neutral pictures. In a pilot study, 291 Chinese participants were asked to rate the arousal and valence of all 704 IAPs pictures [see the Method section of Zhang and Zhou (24, 25)]. In this study, the means and SDs of the arousal and valence ratings for the three picture sets were as follows: negative pictures (arousal: M = 5.75, SD = 0.45, valence: M = 1.99, SD = 0.33), positive pictures (arousal: M = 5.72, SD = 0.45, valence: M = 7.25, SD = 0.38), and neutral pictures (arousal: M = 4.21, SD = 0.27, valence: M = 5.10, SD = 0.45) (see the Supplementary Material). One-way ANOVA was examined for valence and arousal, respectively. The main effect of valence was significant, *F*(2, 122) = 2,641.52, *p* < 0.001, $\eta_{p}^{2}=0.98$. The *post hoc* test showed that positive pictures, negative pictures, and neutral pictures were significantly different in valence (*p*s < 0.001). The main effect of arousal was significant, *F*(2, 122) = 321.66, *p* < 0.001, $\eta_{p}^{2}=0.84$. The *post hoc* test showed that emotional pictures differed significantly from neutral pictures in arousal (*p*s < 0.001). However, there was no significant difference between positive pictures and negative pictures in arousal (*p* > 0.05).

### Procedure

After completing Beck depression inventory (BDI) (26), Beck anxiety inventory (BAI) (27), and Positive and Negative Affect Schedule (PANAS) (28), participant was seated in a comfortable chair facing a computer screen at a distance of 70 cm. Electrodes were attached. First, recording of resting EEG was abstained for each participant. Participants were asked to complete a 2-min resting task. Research showed that time of recording on asymmetry scores did not impacted the internal consistency (29), and that time of 2-min recording was similarly reliable to time of 8-min recording for resting EEG asymmetry (30–32). Then, participants were asked to complete the emotion task in which participants passively viewed emotional pictures. Participants viewed 186 items total, which were divided into three blocks based on the picture types (positive pictures, negative pictures, and neutral pictures). There were 62 trials for each block. In each trial, a blank screen was presented for 300 ms, and then emotional picture was presented for 1,000 ms following a randomized interstimulus interval of 2,200–2,700 ms.

### EEG Recording and Analysis

Electroencephalography data were recorded using the NeuroScan recording and analysis system with 40 Ag/AgCl electrodes positioned according to the International 10–20 system. EEG signals were acquired by a DC model with a sampling rate of 1,000 Hz and a bandwidth of 100 Hz. By electrodes placed above and below the outer orbits of the left eye, vertical and horizontal electrooculograms (EOGs) were recorded. During EEG recording, the electrode on the left mastoid served as a reference. For all of the electrodes, impedance was kept under 5 kΩ.

#### EEG Analysis

The EEG asymmetry measures were taken from the 2-min resting task, and the asymmetry scores were calculated from both eyes open condition and eyes closed condition. EEG data were re-referenced offline to Cz. All data were inspected visually, to eliminate intervals in which ocular or muscle artifacts occurred. Power spectra were derived by means of a fast Fourier transform with a Hamming window (epoch length 1 s, 50% overlap) for resting task for each participant. For consistency with previous research (33, 34), we focused on the alpha band (8–13 Hz) in the frontal electrodes F3 and F4. A laterality coefficient (LC) indexing relative left versus right sided activation was used. EEG LC were computed as follows: LC = ((*L* − *R*)/(*L* + *R*)) × 100. Positive values indicated higher alpha activity in the left than in the right hemisphere (4). There was a long tradition for the calculation of LC in laterality research (35). EEG studies showed that there is a perfectly correlation between LC and another common metric (ln *L* − ln *R*) (36, 37).

#### ERP Analysis

The ERP measures were taken from the emotion task. ERP averages were locked to the onset of the picture from 100 ms before picture onset until 1,000 ms after. For each participant, the average number of artifact-free trials of each ERP grand average was 50, and the minimum number was 45. EEG data were re-referenced offline to the averaged mastoids and were filtered using a 0.05–30 Hz bandwidth. Vertical and horizontal EOGs were filtered out based on independent components analysis used in ERP studies. The rejection criterion of artifact was amplitude of ±100 µV. EEG data were averaged by time window from 100 ms pre-picture cues to 1,000 ms post-picture cues. Based on previous studies (21–23), there were two time windows for mean amplitude measurements, including the P300 (300–500 ms) and the LPP (500–1,000 ms). The present studies computed the centro-parietal region by averaging across electrodes (CP3, CPz, CP4, P3, and Pz). Research showed P300 and LPP were typically observed in centro-parietal electrodes (21, 22, 38, 39).

### Data Analysis

According to a median split of frontal EEG alpha asymmetry scores during the 2-min resting task, individuals can be divided into two groups: left active group vs. right active group. Previous studies showed that this was a valid method for separating the impact of individual differences in frontal EEG alpha asymmetry at resting conditions (4, 40). To investigate the influence of frontal EEG alpha asymmetry at resting conditions on picture perception, repeated measures ANOVAs were performed including valence (negative, positive, and neutral) as within subject variable and group (left vs. right) as between subject variable for LC, P300 amplitude, and LPP amplitude, respectively. A Greenhouse–Geisser correction was applied to all ANOVAs when necessary. The significance levels were set at 0.05.

## Results

Participants were subdivided into groups based on the median of frontal EEG alpha asymmetry scores at F3/F4 electrodes during the 2-min resting task (left active group: 18, −10.64 ± 8.12; right active group: 18, 9.15 ± 7.14). The independent samples *t*-test showed that there were no significant differences between left active group and right active group for age, BAI, BDI, PANAS (negative emotion), and PANAS (positive emotion), respectively (see Table 1).

**Table 1:** The description results of age, Beck anxiety inventory (BAI), Beck Depression Inventory (BDI), and Positive and Negative Affect Schedule (PANAS) for left active group and right active group.

Types	Left active group (*N* = 18)	Right active group (*N* = 18)	*t* (*p*)
Age	21.89 ± 2.54	23.00 ± 2.83	−1.24 (0.22)
BAI	31.94 ± 8.86	29.11 ± 5.64	1.15 (0.26)
BDI	13.56 ± 9.84	12.00 ± 7.37	0.54 (0.60)
PANAS (negative emotion)	19.39 ± 7.75	18.61 ± 7.01	0.32 (0.75)
PANAS (positive emotion)	22.22 ± 7.27	23.89 ± 7.32	−0.69 (0.50)

### EEG Results

**Figure 1:**
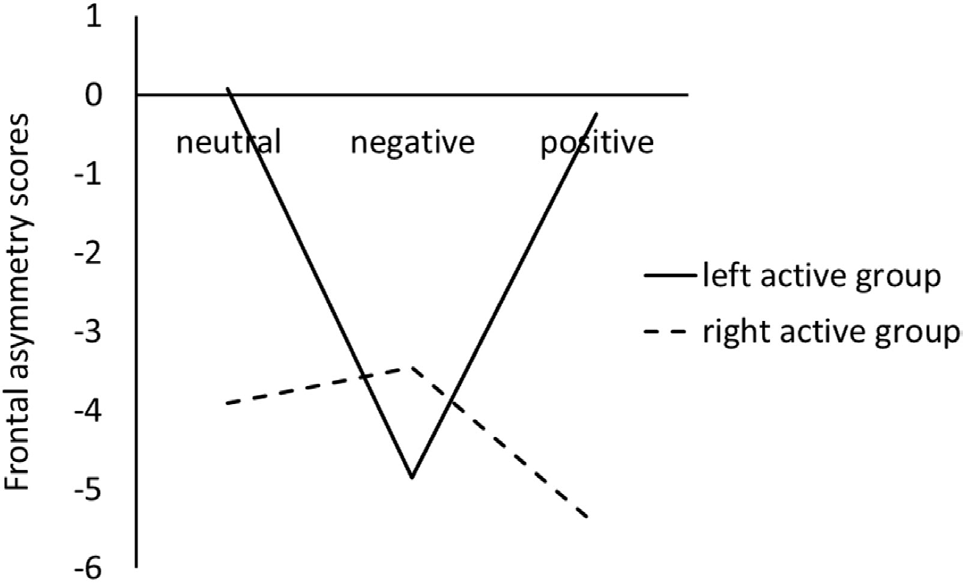
Frontal electroencephalography alpha asymmetry scores during viewing negative, positive, and neutral pictures for left active group and right active group.

Figure 1 showed the difference between left active group and right active group on frontal EEG alpha asymmetry scores for the three pictures stimuli.

For the LC, the ANOVA results revealed that there were not significant main effects of valence, *F*(2, 68) = 1.44, *p* = 0.24, $\eta_{p}^{2}=0.04$, and group, *F*(1, 34) = 0.11, *p* = 0.74, $\eta_{p}^{2}<0.01$. However, a significant valence × group interaction was obtained, *F*(2, 68) = 3.54, *p* = 0.04, $\eta_{p}^{2}=0.09$; simple effect analysis suggested that for left active group, negative pictures induced smaller LC than positive (*p* = 0.02) and neutral (*p* = 0.02) pictures, whereas there was no significant difference between neutral pictures and positive pictures; for the right active group, there were not significant differences among negative, positive, and neutral pictures (see Figure 1).

### ERP Results

**Figure 2:**
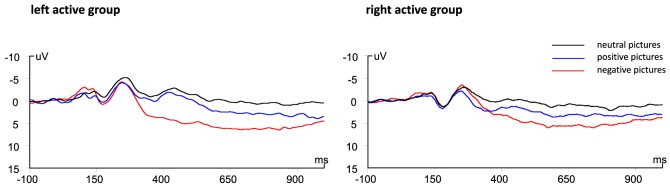
Grand average event-related potentials (ERPs) showing the average across centro-parietal electrodes. Left: ERPs for neutral, negative, and positive images collapsed across left active group. Right: ERPs for right active group.

Figure 2 showed the grand average ERPs during viewing negative, positive, and neutral pictures for left active group and right active group.

**Figure 3:**
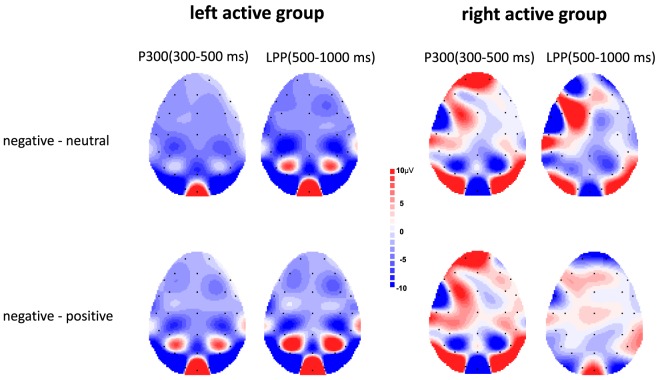
Topographic maps depicting voltage differences for negative minus neutral pictures and negative minus positive pictures in the time range of P300 (300–500 ms) and late positive potential (LPP) (500–1,000 ms) for left active group (left) and right active group (right).

Figure 3 showed voltage differences for negative minus neutral pictures and negative minus positive pictures in the time range of P300 (300–500 ms) and LPP (500–100 ms) for left active group and right active group.

#### P300

The ANOVA results revealed a significant main effect of valence, *F*(2, 68) = 17.00, *p* < 0.001, $\eta_{p}^{2}=0.33$, indicating negative pictures induced greater P300 amplitude than positive (*p* < 0.01) and neutral (*p* < 0.001) pictures (see Figure 2). There was no significant main effect of group, *F*(1, 34) = 0.08, *p* = 0.78, $\eta_{p}^{2}<0.01$. However, a significant valence × group interaction was also obtained, *F*(2, 68) = 4.67, *p* = 0.02, $\eta_{p}^{2}=0.12$. Simple effect analysis suggested that for left active group, negative pictures induced larger P300 amplitude than positive (*p* < 0.001) and neutral (*p* < 0.001) pictures, whereas there was no significant difference between neutral pictures and positive pictures (see Figures 2 and 3); for right active group, there were no significant differences among negative, positive, and neutral pictures.

#### Late Positive Potential

The ANOVA results revealed a significant main effect of valence, *F*(2, 68) = 10.95, *p* < 0.001, $\eta_{p}^{2}=0.24$, indicating negative pictures induced greater LPP amplitude than positive (*p* = 0.02) and neutral (*p* < 0.001) pictures, and positive pictures induced greater LPP amplitude than neutral pictures (*p* = 0.02) (see Figure 2). There are no significant main effect of group, *F*(1, 34) = 0.41, *p* = 0.53, $\eta_{p}^{2}=0.01$, and the interaction between group × valence was not significant, *F*(2, 68) = 1.71, *p* = 0.19, $\eta_{p}^{2}=0.05$.

## Discussion

This study showed that frontal EEG alpha asymmetry at resting conditions predicted the evaluation of emotional picture stimuli. Left active individuals had smaller frontal EEG alpha asymmetry scores to view negative than to view positive and neutral pictures, whereas right active individuals had similar frontal EEG alpha asymmetry scores when viewing negative, positive, and neutral pictures. Furthermore, the study showed a larger P300 to negative pictures than to positive and neutral pictures for left active individuals; however, there were no significant ERP differences to negative, positive, and neutral pictures for right active individuals.

The EEG finding that there was different neural performance between left active individuals and right active individuals was in line with our expectation. That is, frontal EEG alpha asymmetry at resting conditions can regulate the frontal alpha EEG asymmetry response to emotional picture. This was consistent with previous studies. Coan and Allen (41) showed that individuals with left frontal EEG alpha asymmetry at resting conditions reported increased emotional experience and increased the emotional intensity, compared with individuals with right frontal EEG alpha asymmetry. Kline et al. (40) and Papousek et al. (4, 10) showed that individuals with left frontal EEG alpha asymmetry at resting conditions exhibited EEG asymmetry changes when experiencing emotional stimulation, whereas individuals with right frontal EEG alpha asymmetry appeared unresponsive to emotional stimulations. Thus, the results showed there was flexible response when viewing emotional pictures for left active individuals, whereas there was inflexible response for right active individuals.

Especially, the ERP findings showed that compared with right active individuals, left active individuals had larger P300 effect to negative pictures than to positive and neutral pictures. Emotional ERP modulations can be explained by the “negativity-bias” effect. Research showed that there were valence ERP effects that indicated greater P300 magnitude to negative pictures than to neutral pictures or positive pictures (15–17, 42). The “negativity-bias” effect emphasized the intrinsic relevance for negative stimuli (43, 44) and was considered to response to attention processing (45, 46). Thus, emotional P300 effects may reflect the rapid attention to the threatening stimuli, and then improve individual processing efficiency (47). According to this view, for individuals with left frontal EEG alpha asymmetry, attention can be automatically directed to a threatening stimulus from an evolutionarily adaptive perspective. However, for individuals with right frontal EEG alpha asymmetry, the “negativity-bias” effect disappeared, which indicated that they did not response to a threat and did not adapt the environment quickly.

The neuro-laterality models of affect and psychopathology provided a theoretical basis for differential involvement of left and right frontal cortical regions in emotional processing. The laterality models assume the left and right frontal cortical hemispheres to be differentially involve in processes modulating affective responses to emotional challenges (3, 36). It has been proposed that greater left frontal EEG activity at baseline is associated with greater affective flexibility as compared to asymmetry in favor of the right hemisphere (4). Our findings support the neuro-laterality models of affect and psychopathology. That is, individual frontal EEG alpha asymmetry at resting conditions can predict the response to emotional stimuli.

This study is based on the view that frontal EEG alpha asymmetry at resting conditions reflects a personal trait to react emotionally. The study demonstrates that frontal EEG alpha asymmetry predicts the evaluation of emotional picture stimuli. On the one hand, frontal EEG alpha asymmetry at resting conditions may moderate the transient EEG asymmetry response to emotional picture stimuli. On the other hand, compared to right active individuals, left active individuals put more attentional resources to negative pictures than to positive and neutral pictures.

## Ethics Statement

This study was carried out in accordance with the recommendations of the local Ethics Committee of Henan University with written informed consent from all participants. All participants gave their written informed consent in accordance with the Declaration of Henan University. The protocol was approved by the local Ethics Committee of Henan University.

## Author Contributions

All the authors took part in planning the theoretical and conceptual basis for the study. All the authors took part in critically reviewing and editing the manuscript.

## Conflict of Interest Statement

The authors declare that the research was conducted in the absence of any commercial or financial relationships that could be construed as a potential conflict of interest.
