# Supplementary material for: The Functional Role of Individual-Alpha Based Frontal Asymmetry in the Evaluation of Emotional Pictures: Evidence from Event-Related Potentials
Source: Front Psychiatry. 2017 Sep 27;8:180. doi: 10.3389/fpsyt.2017.00180 (PMC5623932; doi:10.3389/fpsyt.2017.00180)
Supplement: Supplementary file 1 [file Data_Sheet_1.docx]

Appendix

Pictures from the International Affective Picture System (IAPS) Used in the Experiment

**Neutral pictures**

2200 2210 2215 2221 2270 2280 2320 2372 2383 2480 2485 2495 2514 2518 2570 2575 2580 2600 2749 5500 5510 5520 5531 5534 5875 5991 6150 7000 7002 7004 7006 7009 7010 7020 7025 7030 7031 7034 7035 7040 7050 7060 7080 7090 7095 7096 7100 7130 7140 7150 7170 7175 7180 7183 7184 7185 7186 7187 7190 7205 7211 7217

**Positive pictures**

1340 1440 1460 1463 1600 1601 1603 1604 1710 1750 1812 1920 1999 2030 2040 2050 2058 2070 2091 2209 2216 2260 2303 2310 2340 2341 2345 2391 2501 2530 2550 2655 2660 2791 4614 4617 4641 4770 5000 5001 5010 5201 5450 5470 5621 5626 5628 5750 5760 5779 5780 5800 5831 5849 5891 7220 7230 7270 7282 7283 7289 7402

**Negative pictures**

1040 1050 1052 1070 1111 1113 1120 1280 2800 3000 3010 3015 3030 3051 3053 3060 3061 3062 3064 3071 3080 3100 3102 3110 3120 3130 3140 3150 3160 3168 3170 3250 3261 3266 3301 3350 3400 3530 3550 6010 6300 6370 6550 6570 6831 8230 9042 9140 9265 9300 9320 9400 9405 9410 9420 9430 9433 9480 9570 9571 9584 9921
